# Epoxy/Graphene Nanoplatelet (GNP) Nanocomposites: An Experimental Study on Tensile, Compressive, and Thermal Properties

**DOI:** 10.3390/polym16111483

**Published:** 2024-05-23

**Authors:** Mahmuda Akter, Huseyin Ozdemir, Kadir Bilisik

**Affiliations:** 1 Nano/Microfiber Preform Design and Composite Laboratory, Department of Textile Engineering, Faculty of Engineering, Erciyes University, Talas, Kayseri 38039, Turkey; mahmuda.akter.mitu88@gmail.com; 2 Department of Apparel Engineering, Faculty of Fashion Design and Apparel Engineering, Bangladesh University of Textiles, Tejgaon, Dhaka 1208, Bangladesh; 3 Textile and Fashion Design Department, Faculty of Fine Arts, Gaziantep University, Gaziantep 27310, Turkey; hozdemir@gantep.edu.tr; 4 Nanotechnology Application and Research Centre (ERNAM), Erciyes University, Talas, Kayseri 38039, Turkey

**Keywords:** graphene nanoplatelets (GNPs), epoxy nanocomposites, tensile properties, fracture surface, damage tolerance

## Abstract

This paper presents an experimental investigation of nanocomposites composed of three ratios of epoxy/graphene nanoplatelets (GNPs) by weight. The 0.1, 0.2, and 0.3 wt.% specimens were carefully manufactured, and their mechanical and thermal conductivity properties were examined. The tensile strength and modulus of epoxy/GNPs were enhanced by the large surface area of graphene nanoplatelets, causing crack deflection that created new fracture fronts and friction because of the rough fracture surface. However, the compressive strength was gradually reduced as GNP loading percentages increased. This was probably due to severe plastic yielding on the epoxy, leading to catastrophic axial splitting caused by premature fractures. Furthermore, the highest thermal conductivity was 0.1283 W/m-K, representing a 20.92% improvement over neat epoxy (0.1061 W/m-K) when 0.3 wt.% GNPs were added to the epoxy. This was because of efficient heat propagation in the GNPs due to electron movement through percolative paths. The tensile failure mode in epoxy/GNP nanocomposites showed a few deflected and bifurcated rough cracks and brittle, dimple-like fractures. Contrarily, compressive failure mode in GNP-added epoxy showed plastic flexural buckling and brittle large-axial splitting. The epoxy/GNP nanocomposites were considered a damage-tolerant material.

## 1. Introduction

Materials, especially nanocomposites and fiber-based nanocomposites [1,2,3,4], are today important across all technological domains within the sectors of health, electronics, aeronautics, energy, and sensors [5,6]. It is crucial to consistently enhance current materials and create novel ones with improved features and versatility to enable their use in diverse applications. Polymer nanocomposites involve blending polymer matrices with diverse filler materials, which may be organic or inorganic and possess at least one dimension within the nanometer scale. Innovative polymer nanocomposites play a big part in developing processability because they combine toughness with the ability to do practical electrochemical processes [7]. Nanocomposites have exceptional properties compared to conventional composite materials because of a ratio where surface area is high relative to volume [8]. The usage of nanofillers has been thoroughly studied in the past and has demonstrated the potential enhancement of many polymer features, including their mechanical [9], thermal, and tribological properties [10].

Carbonaceous nanofillers such as graphene and carbon nanotubes (CNTs) exhibit significant promise owing to their exceptional structural and functional characteristics compared to alternative nanofillers [11,12]. The main problem with polymer nanocomposites is that phase separation [13] causes nanoparticles and nanosheets to stick together (agglomeration) and stack at relatively low volume fractions. This presents a challenge for enhancing the performance and mechanical efficiency of the composite, as the nanofillers employed tend to agglomerate due to weak, attractive Van der Waals forces between them. Consequently, this leads to the uneven dispersion of nanofillers within matrices, causing a marginal reduction in the mechanical and thermal properties of the resulting nanocomposites [14]. The most effective enhancement in the properties of the polymer matrix is achieved through the even distribution of nanofillers [15]. Achieving this even dispersion is a critical challenge in nanocomposite processing. Therefore, it is crucial to review currently employed processing techniques for preparing polymer nanocomposites [16].

Polymer nanocomposites incorporating graphene hold promise for advancing next-generation materials, surpassing conventional technologies. Graphene nanoplatelets (GNPs) are increasingly favored in diverse multifunctional, multidimensional [17], and technological applications, such as conductive composites [18,19,20], batteries, sensors, electronics [21], flexible and transparent electrodes for solar cells and displays [22], supercapacitors and energy storage [23,24], electromagnetic interference shielding [25], enhancing biocompatibility and stability of nanocarriers, and reducing size and toxicity [22]. Furthermore, it has been demonstrated that both pristine and functionally graded graphene nanoplatelets exhibit significant promise in augmenting the stiffness of beam, plate, and shell structures subjected to static and dynamic loading [26]. GNPs offer cost effectiveness, a versatile preparation process, and functionalization, with the advantage of lacking toxic metal particles, distinguishing them from other carbon nanomaterials.

Epoxy resin stands out as a widely employed and valuable thermoset polymer due to its cost effectiveness, high weight-to-strength ratio, durability across a broad temperature range, resistance to corrosion, and excellent thermal stability with minimal shrinkage [27]. Its favorable characteristics, including ease of processing and strong adhesion, have led to widespread adoption in various composite applications [28,29,30]. However, the brittleness arising from the high cross-linking density of epoxy remains a notable limitation requiring enhancement [31,32]. Numerous researchers are focused on enhancing the toughness properties of epoxy. An effective strategy to address this challenge is the incorporation of nanofillers into the epoxy polymer [33]. It has been demonstrated that GNPs as nanofillers greatly improve thermoset resins’ mechanical characteristics [34,35].

Researchers have employed various approaches, including filler functionalization [36,37], ultrasonication [38,39], and homogenization [40], to improve the degree of bonding and interfacial adhesion between the epoxy matrix and nanofillers. Combining graphene nanoplatelets with epoxy has introduced a new class of nanocomposites suitable for advanced engineering applications [41]. The properties of polymer/graphene composites are affected by factors such as the filler distribution, filler–matrix adhesion, filler quantity in the matrix, and the quality of graphene. The primary goal of graphene-based polymer nanocomposites is to generate functional materials with higher performance due to the polymer/graphene interphase composition. The transfer of attributes between inter-components is improved when the interphase is stronger. Research has shown that the dispersion of fillers, their size, and the interfacial bonding between reinforcements and the matrix are crucial factors impacting the overall properties of the resulting composites [42].

Epoxy nanocomposites loaded with various numbers of GNPs have been shown to have improved toughness [27,43,44,45], reinforcement [46,47], and thermal properties [48]. Shen et al. [49] found that incorporating 0.5 wt.% graphene sheets into epoxy caused an increase in Young’s modulus at room and cryogenic temperatures. In a study by Yao et al. [50], adding just 0.8 wt.% graphene to epoxy nanocomposites led to a 37% increase in tensile strength and a 63% increase in elongation at break compared to pure epoxy. Rafiee et al. [51] noted a 45% increase in tensile strength by adding 0.125 wt.% graphene platelets to an epoxy resin matrix, compared to neat epoxy. Another research study exhibited that incorporating silane-functionalized graphene oxide into an epoxy matrix notably enhances thermo-mechanical properties, primarily attributed to improved dispersion and more vital interfacial interaction facilitated by covalent bonding between the two materials [52]. It was reported that graphene oxide particles functionalized with hexamethylenediamine decreased the free surface energy at the polymer–nanofiller boundary and improved adhesion [53]. Li et al. [54] demonstrated that introducing amino-functionalized graphene oxide into epoxy enhanced tensile strength, while epoxy-functionalized graphene oxide improved fracture toughness in nanocomposites. Schulte et al. [55] investigated the fracture toughness and failure mechanism of epoxy/GNP composites. They proposed that one of the toughening mechanisms in graphene-added nanocomposites was a crack deflection resulting from higher surface roughness.

The dispersion of graphene and the interfacial bonding within the epoxy matrix, both pivotal factors influencing the performance of these innovative nanocomposites, exert a considerable influence on the advancement and suitability of epoxy/graphene nanocomposites. Therefore, improving the methods for graphene dispersion and tailoring the graphene/epoxy interface is critical to creating improved epoxy/graphene nanocomposites [16]. The extensive surface area of GNP enhances stress transfer from polymer to individual nanoplatelets in polymer/GNP composites. Furthermore, nanocomposites’ properties were significantly influenced by nanoparticle geometry and their placement in the composite [56]. The agglomeration of nanoparticles resulted in a reduction in the elastic properties of the nanocomposites [57]. While processing epoxy/GNP nanocomposites, issues such as agglomeration, nonalignment, heterogeneous dispersion, and graphene damage contributed to some deterioration in the mechanical properties.

Multiple theoretical modeling studies on the GNP-added epoxy composites based on analytic, empirical, and numerical (FEM) methods have been proposed [58,59,60]. Using molecular dynamics simulations, incorporating graphene concentrations ranging from 1% to 3% with a low aspect ratio has been observed to enhance Young’s modulus. The in-plane elastic modulus is improved when graphene is well dispersed in the epoxy medium compared to agglomerated graphene. Furthermore, significant enhancement in cohesive force is achieved by establishing chemical bonding at the graphene–epoxy interface [61]. Continuum modeling is commonly used to analyze nano and micro scales in multiscale composites, while the principle of micromechanical models is applied for modeling meso and macro scales. A stochastic multiscale approach has also been recognized as suitable for predicting elastic properties in multiscale composites subjected to static and dynamic loadings [62].

In this investigation, we employed a straightforward method to explore the combination of graphene nanoplatelets with the low-cost diglycidyl ether of bisphenol-A (DGEBA) epoxy to make epoxy/GNP nanocomposites. Further, their mechanical properties and thermal conductivity were assessed and compared with neat epoxy. The chemical structures and molecular compositions for various epoxy/GNP nanocomposites were analyzed using FTIR and Raman spectroscopy. In addition, we identified their failure modes, especially considering the compressive failure, and explained potential complex toughening mechanisms.

## 2. Materials and Methods

### 2.1. Materials

Graphene nanoplatelets, commercially accessible under the name xGNP^®^-M-25, feature an average particle diameter of 25 μm, a density of around 2.2 g/cm^3^, a thickness of approximately 6–8 nm (comprising nearly 20 graphene layers), and a specific surface area ranging from 120 to 150 m^2^/g [8]. The current study used these nanoplatelets obtained from Rasel Kimya (Sigma Aldrich, XG Sciences, Inc., Lansing, MI, USA) as reinforcing fillers.

The resin used in this study was epoxy (HEXION MGS^®^ LR326, Columbus, OH, USA), and hardener (HEXION MGS^®^ H265, Columbus, OH, USA) was used to produce epoxy/graphene nanoplatelet nanocomposites. The epoxy resin and hardener were procured from Dost Kimya Co., Tuzla/Istanbul, Turkey. The resin, characterized by low viscosity, was a bisphenol-A epoxy resin. MGS LR326, chosen for its high thermo-mechanical properties, was the selected hardener. Per the manufacturer’s recommendation, the recommended mixing ratio of resin to hardener was 100:25, in parts by weight (epoxy: hardener). Table 1 shows the specifications for the epoxy and hardener.

### 2.2. Preparation of Epoxy Composite

**Figure 1:**
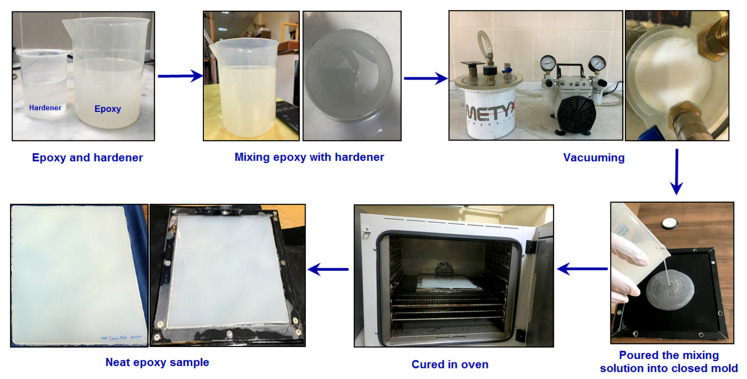
Experimental procedure for neat epoxy nanocomposites.

For comparison purposes, neat epoxy was produced. The mold dimensions (length, width, and thickness) used in this study were 300 mm × 300 mm × 5.5 mm, respectively. The metal mold was coated with Teflon film. Polyurethane grease was applied to the inside surfaces of the closed mold for easy demolding. The required amount of epoxy and hardener was calculated according to mold size and weighted using a digital balance (KERN & Sohn GmbH, model: KERN FCB BK01, Balingen, Germany). After that, epoxy and hardener were mixed gently by hand with a glass rod for 10 min. Then, the mixture was vacuumed in a vacuum chamber (Gardner Denver Thomas GmbH-WELCH, model: 2522C-02, Ilmenau, Germany) for 30 min under a pressure of 0.1 MPa to remove any air bubbles. The mixed epoxy polymer matrix was then ready for molding. Then, the resin blend was poured into a sealed mold, exposing the upper surface. The mold was put into an oven (BINDER GmbH, model: FD115, Tuttlingen, Germany) for curing at a temperature of 120 °C for 6 h. Afterward, the mold was permitted to cool within the press until the temperature reached 40 °C. Later on, the sample was collected from the oven. The closed mold was demolded to remove the sample. Finally, the plate was kept in a standard laboratory atmosphere for three days. The neat epoxy composite plate was then ready for testing and characterization. Figure 1 shows the actual experimental procedure for neat epoxy nanocomposites.

### 2.3. Preparation of Epoxy/Graphene Nanoplatelet Composite

**Figure 2:**
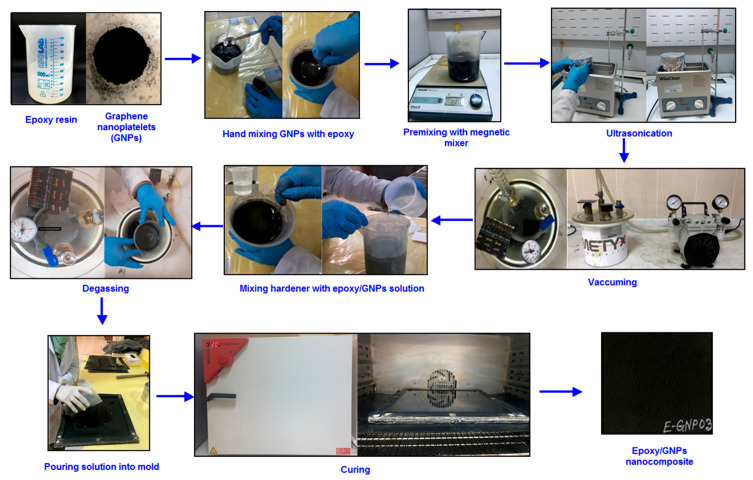
Experimental procedure of epoxy/GNP nanocomposites.

Three ratios (0.1 wt.%, 0.2 wt.%, and 0.3 wt.%) were selected as nanofiller loading percentages to produce epoxy/GNP nanocomposites. The mold specifications in the nanocomposite were identical to those in the neat epoxy. The mold was Teflon-coated for easy demolding. Polyurethane grease was applied to the inside surfaces of the closed mold for easy demolding. The necessary quantity of epoxy and hardener was determined according to the mold size. Subsequently, the GNPs were computed (relative to the epoxy weight) and weighed using a digital balance (Shimadzu Corp., model: AUX320, Kyoto, Japan). The GNPs were gradually added in small increments to the epoxy and mixed gently with a glass rod for 10 min to prevent possible GNP agglomeration. The GNPs were pre-mixed with epoxy resin using a magnetic mixer (Wisestir^®^, Witeg Labortechnik GmbH, Wertheim, Germany) at 220 rpm for five minutes. The matrix/GNP solution underwent ultrasonic mixing in a bath (200 W, 40 KHz, WUC-A03H/Wise Clean^®^, DAIHAN Inc., Seoul, Republic of Korea) at 25 °C for two hours. The resulting mixture was then vacuumed in a vacuum chamber for 30 min under a pressure of 0.1 MPa to eliminate air bubbles. The epoxy/GNP resin mixture received the addition of hardener (epoxy and hardener ratio = 4:1) and was gently stirred with a glass rod for 10 min. Once again, the mixture was vacuumed for 15 min under a pressure of 0.1 MPa. The epoxy/GNP matrix was then ready for molding. Next, the epoxy/GNP resin blend was poured into a sealed mold with the top surface exposed. The mold underwent curing in an oven at 120 °C for six hours. Subsequently, the mold was allowed to cool gradually in the oven until the temperature reached 40 °C. Following this, the sealed mold was demolded to extract the final plate. It was kept in a standard laboratory atmosphere for three days. The E-GNP01, E-GNP02, and E-GNP03 composite plates were then ready for testing and characterization (Table 2). Figure 2 shows the experimental procedure for neat epoxy nanocomposites.

### 2.4. Composite Testing

#### 2.4.1. Tensile Test

**Figure 3:**
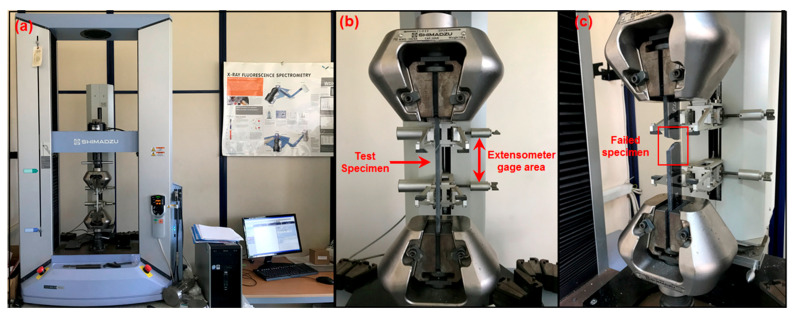
A tensile test sample of epoxy/GNP nanocomposites in a universal testing machine. (**a**) Before the test, (**b**) during the test and (**c**) after the test.

The tensile strength characteristics of composite structures were assessed following the ASTM D3039-14 standard [63]. The tensile strength test used the Shimadzu Corp. Model: AG-XD 50 (Kyoto, Japan) universal testing instrument at Erciyes University Technology Research and Application Center (ERUTAUM). Test results were obtained via Trapezium^®^ software, version 1.1.2. Figure 3 shows the tensile strength tester with test coupons. The specimen dimensions were selected following ASTM D3039-14. The test dimensions were 250 mm (length) × 25 mm (width) with an average 6.11 mm thickness for the epoxy plate. For tensile testing of epoxy/GNP nanocomposites, an extensometer was affixed to the testing machine specimens to ensure accurate strain measurement. The samples were stored in the mechanical testing room at a constant temperature of 23 ± 0.1 °C and humidity of 50 ± 5% for three days before mechanical testing. The universal testing machine, equipped with a 50 kN load cell, maintained a crosshead speed of 2 mm/min. A least three composite samples were evaluated for each loading, and the results were averaged. The ultimate tensile strength was calculated using Equation (1).

F^tu^ = F^max^/A (1)

Here, F^max^ denotes the maximum force before failure (N), A represents the average cross-sectional area (mm^2^), and F^tu^ stands for the ultimate tensile strength. The tensile stress and strain at each specified data point can be derived using Equations (2) and (3). The tensile modulus in the elastic region of the stress–strain curve is expressed by Equation (4).

σ_i_ = F_i_/A (2)

ε_i_ = δ_i_/L_g_ (3)

*E*_chord_ = Δσ/Δε (4)

Here, σ_i_ signifies the tensile stress at the ith data point (MPa), F_i_ is the force at the ith data point (N), ε_i_ denotes the tensile strain at the ith data point (με), δ_i_ is the extensometer displacement at the ith data point (mm), L_g_ is the extensometer gauge length (mm), E_chord_ represents the tensile modulus of elasticity (GPa), Δσ indicates the change in applied tensile stress (MPa), and Δε represents the variance between the two strain points. Figure 3 depicts the tensile test sample of epoxy/GNP nanocomposites before and after testing.

#### 2.4.2. Compression Test

**Figure 4:**
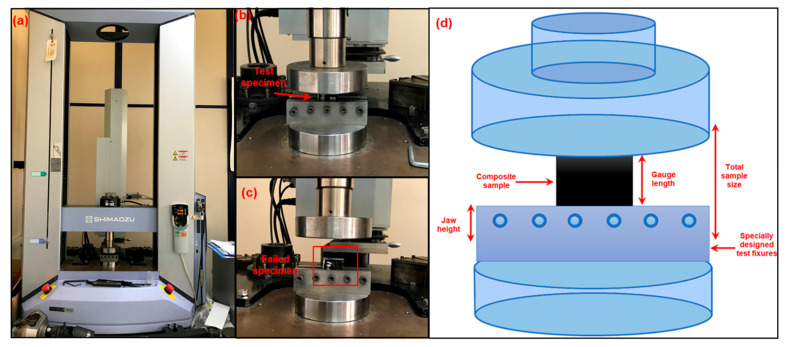
Compressive test for epoxy/GNP nanocomposites. (**a**) Universal testing machine with sample and (**b**) test specimen under fixture before test and (**c**) after test. (**d**) In-house-designed fixture schematic.

This study aimed to explore the compressive behavior of polymer matrix nanocomposites. Compressive strength tests followed the modified ASTM D695-08 standard [64]. Compressive strength testing was accomplished using a Shimadzu Corp. Model: AG-XD 50 (Kyoto, Japan) model universal testing machine. Compression tests were conducted with a specially designed in-house fixture. The test dimensions of the samples were 38 mm (length) × 25 mm (width) × 6.11 mm (thickness) considering the ASTM D695-08 standard. Figure 4 depicts a schematic diagram of the compressive test experimental setup. The apparatus was fitted with a 50 kN load cell, and the crosshead speed was configured at 1 mm/min. A minimum of three composite samples for each plate were subjected to testing, and the outcomes were averaged. The specimen’s compressive strength is determined by dividing its most significant compressive load by its initial cross-sectional area. To calculate the compressive strength, Equation (5) is used, as follows:

F_cu_ = F_max_/A (5)

Here, F_cu_ denotes the compressive strength (MPa), F_max_ represents the maximum force before failure (N), and A signifies the cross-sectional area at the test section (mm^2^).

#### 2.4.3. Thermal Conductivity Test

**Figure 5:**
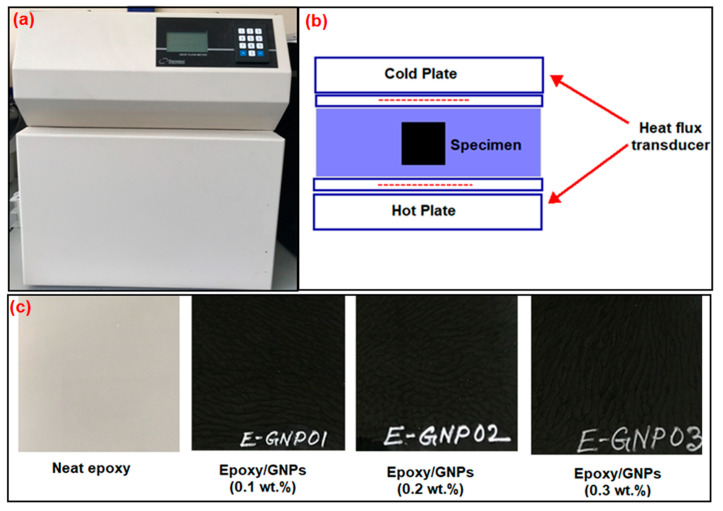
(**a**) Actual image of heat flow, (**b**) schematic diagram and the concept of heat flow and temperatures, and (**c**) thermal conductivity test samples.

The thermal conductivity of each composite sample was assessed using a heat flow meter (Thermtest, model: HFM-100, New Brunswick, CA, USA), as illustrated in Figure 5, following the ASTM Standard C518-02 [65]. The sample size was 100 mm (length) × 100 mm (width) to measure the thermal conductivity. A specimen was positioned between two heating–cooling plates, and the upper plate descended to make contact with the sample, driven by stepper motors at each corner, as depicted in Figure 5a,b. A conventional pressure was exerted, or a user-defined specimen thickness controlled plate contact with the test specimen. Figure 5c shows the specimen size required for thermal conductivity. Individual optical encoders controlled the stepper motors to measure sample thickness (L) to the nearest 0.1 mm. The upper plate could sense and adjust for specimens with surface differences due to integrated logic between stepper motors, optimizing plate–specimen contact for measurements. Each plate incorporated a heat flux sensor for measuring the heat flow (Q/A) resulting from the temperature differential (T) between the top and bottom plates at regular intervals until a steady-state heat flow was achieved. Subsequently, the composite heat flux was utilized to compute thermal conductivity (λ) in accordance with Fourier’s law.

λ = S × E × (L/ΔT)(6)

Here, λ represents thermal conductivity (W/(mK)), S is the calibration factor of the heat flux transducer ((W/m^2^)/V), E stands for the heat flux transducer output (V), L denotes the separation between the hot and cold plate assemblies during testing (m), and ΔT indicates the temperature difference across the specimen (°K).

### 2.5. Analysis and Characterizations

The fracture surface morphologies of epoxy/GNP composites were analyzed using a field emission scanning electron microscope (FESEM, Carl Zeiss GmbH, Model: ZEISS GeminiSEM500, Oberkochen, Germany) under standard laboratory conditions. An FT-IR spectrometer (Perkin Elmer^®^ Spectrum 400, PerkinElmer Inc., Waltham, MA, USA) was employed to capture infrared spectra in absorbance mode for identifying chemical modifications in the composite structures. The analysis covered a wavenumber range of 4000–400 cm^−1^ with a characteristic resolution of 2 cm^−1^.

In Raman analysis, the IG/ID intensity ratio is a widely employed metric to assess the level of imperfection or disorder within a structure. Precisely, this ratio gauges the intensity ratio of the G-mode to the D-mode [66]. The “G” band typically corresponds to sp2 hybridized carbon atoms in the nanotube wall, while the “D” band arises from structural defects and sp3 hybridized carbon atoms. The intensity of the “D” band is inversely related to the quality of the nanotube [67]. The composite panels’ chemical structures and molecular compositions were analyzed using a Raman Imaging Microscope–WITec (alpha300, WiTec GmbH, Ulm, Germany) with a laser wavelength of 532 nm.

The surface roughness of failed epoxy/GNP nanocomposites was measured to identify the possible contribution of nanoplatelet particles to the tensile and compression strength of the structure by using contact profilometers (Bruker, model: DektakXT, Vision 64 MAP^TM^ application software, Billerica, MA, USA). Surface roughness was assessed using Gwyddion (http://gwyddion.net/), an open-source software designed for Statistical Parametric Mapping (SPM) data analysis [68]. Additionally, the densities of epoxy/GNP composites were determined following the ASTM D792-08 guidelines [69].

## 3. Results and Discussion

### 3.1. Surface Morphology and Structure

#### 3.1.1. FTIR Spectroscopy Result

**Figure 6:**
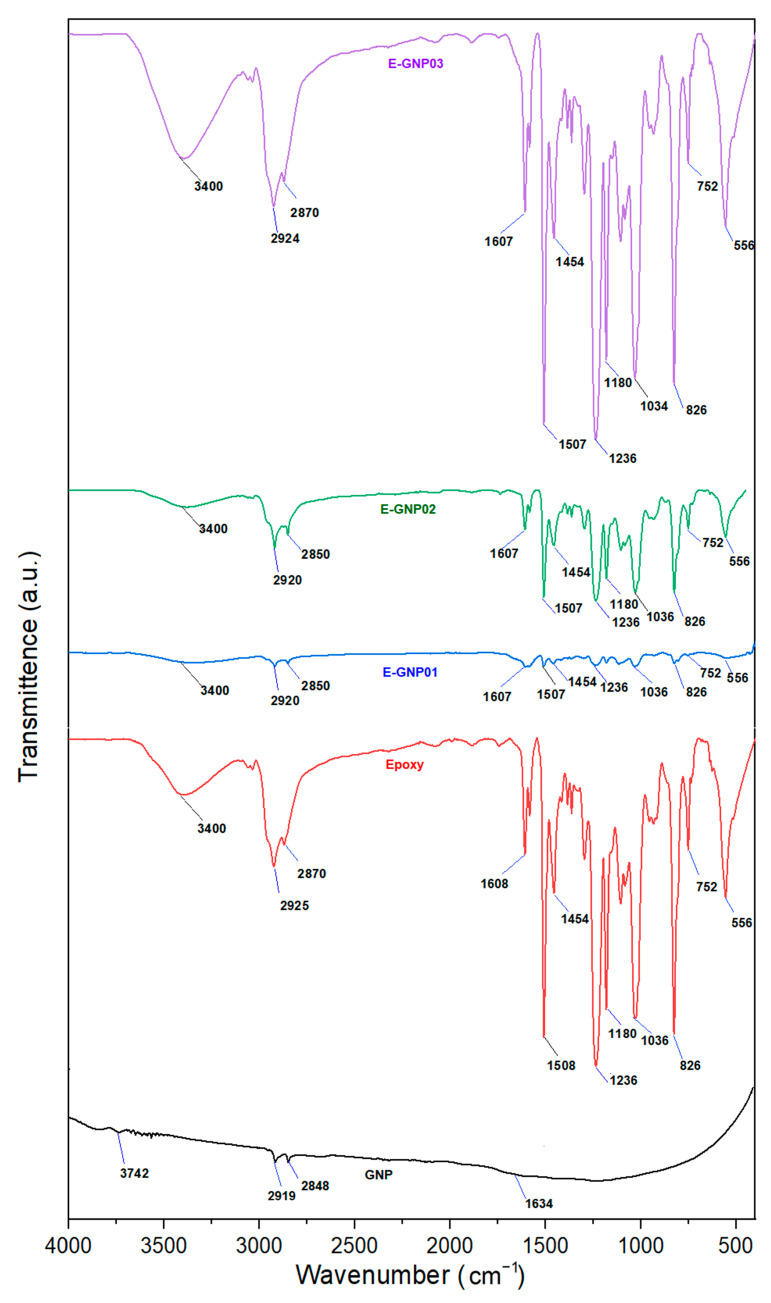
Fourier transform infrared spectra of graphene nanoplatelets, pristine epoxy, and GNP-added epoxy composites.

The Fourier transform infrared (FTIR) spectrum measurements were utilized to identify the functional groups within the materials and gain insight into the interaction between GNPs and epoxy molecules. Table 3 shows the FTIR spectra identified for epoxy and GNP-added epoxy composites. The FTIR spectra of graphene nanoplatelets, pristine epoxy, and epoxy/GNP composites are presented in Figure 6. As depicted, the infrared spectra of GNPs reveal minor peaks at approximately 1550 cm^−1^ and 1650 cm^−1^, indicative of a C=O stretching vibration from the carboxylic group and the involvement of skeletal graphitic carbon atoms. The FTIR spectra reveal prominent absorption peaks related to both the neat epoxy polymer and EGNP. Peaks at 2928 cm^−1^ and 2868 cm^−1^ are associated with the valence –CH vibrations of the epoxy ring and the stretching –CH vibrations of the –CH_2_ group of aromatic rings, respectively. Additionally, the symmetrical –CH stretching vibrations of the –CH_3_ functional group are identified at the peak around 2868 cm^−1^. The IR spectra of neat epoxy exhibit prominent peaks at: (i) 1608 cm^−1^, 1508 cm^−1^, and 1454 cm^−1^, corresponding to a C–C stretching vibration of aromatic rings; (ii) 1297 cm^−1^, associated with an asymmetrical deformation of –CH_2_ groups; (iii) vibrational frequencies at 1235 cm^−1^, 1180 cm^−1^, 1035 cm^−1^, attributed to asymmetrical aromatic and aliphatic C–O stretching of the epoxy; (iv) 826 cm^−1^, assigned to –CH out-of-plane deformation in aromatic and epoxide ring vibrations; and (v) 555 cm^−1^, denoting the frequency of aromatic groups.

The results indicate that the monomer effectively underwent polymerization with the hardener, creating a solid epoxy resin. The IR spectrum of an epoxy composite with a lower concentration of GNPs closely resembled that of neat epoxy, with the leading bands shifting wave numbers by 5–10 cm^−1^. This shift suggests the formation of composites through physical or Van der Waals interactions between epoxy molecules and GNPs. It highlights the anticipated bonding interaction between epoxy and its reinforced mixed nano-filler GNPs.

#### 3.1.2. Raman Spectroscopy Result

**Figure 7:**
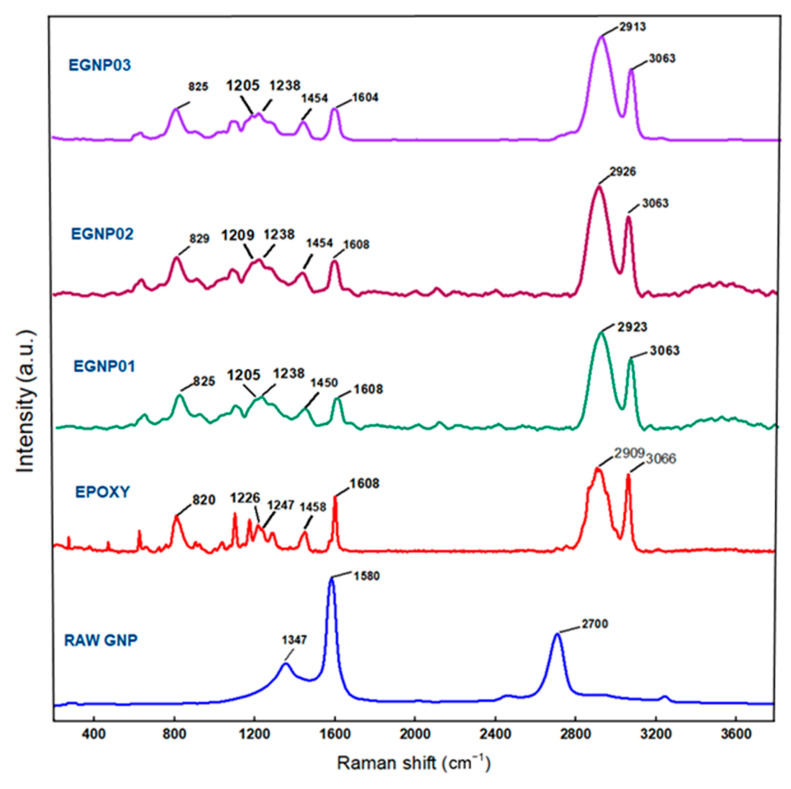
Raman spectra of graphene nanoplatelets, pristine epoxy, and GNP-added epoxy composites.

Raman spectroscopy is highly effective in verifying the presence of GNPs in composite materials. The epoxy resin fingerprint is depicted in Figure 7, with the Raman shift corresponding to epoxide vibration between 1280 cm^−1^ and 1230 cm^−1^. In this study, the measurement of epoxide vibration was recorded at 1247 cm^−1^ [70]. The Raman peak at 826 cm^−1^ is attributed to -CH wagging vibrations, while resin backbone vibrations are evident at 1141 cm^−1^, 1205 cm^−1^, and 1454 cm^−1^ [71]. Additionally, the double peak around 1608 cm^−1^, corresponding to the C=C vibration of the aromatic ring and CHx vibration in the band near 2900 cm^−1^, affirms the aromatic nature of the bearing aromatic group (where X = functional groups). The introduction of GNPs induces a notable G-band at 1580 cm^−1^, signifying graphitized sp2-hybridized carbon. The weak D-band at 1347 cm^−1^ indicates structural defects in the GNPs, and a 2D peak at 2700 cm^−1^ is observed. The G-peak results from the double-generated center E2g vibration stretching [7], with the D-band arising from this phenomenon.

A noticeable shift in the epoxy peak is evident upon adding GNPs. The epoxy breathing peak at 1247 cm^−1^ undergoes a shift to lower frequencies, approximately 1226 cm^−1^, 1238 cm^−1^, and 1238 cm^−1^ in sample compositions of 0.1 wt.% EGNP01, 0.2 wt.% EGNP02, and 0.3 wt.% EGNP03, respectively, indicating interference from GNPs. The introduction of GNPs also results in the emergence of a prominent G-band at 1580 cm^−1^, denoting graphitized sp2-hybridized carbon. Furthermore, a weak D-band at 1347 cm^−1^, indicating structural defects in the GNPs, and a 2D peak at 2700 cm^−1^ are observed.

### 3.2. Mechanical Properties

#### 3.2.1. Density of Nanocomposites

Density (ρ) values for both neat epoxy and various epoxy/GNP nanocomposites were obtained by weighing the composites in water and air, utilizing a specific gravity kit (Precisa Gravimetrics AG, Model:Precisa XB 220A, Dietikon, CH). The average densities of neat epoxy (E) and the E-GNP01, E-GNP02, and E-GNP03 composite samples are detailed in Table 4, categorized by their respective codes and their standard deviation (SD) and coefficient of variation (CV%) values. The average density of all epoxy/GNP nanocomposites exhibited insignificant variations. The uniform dispersion and homogeneous distribution of graphene nanoplatelets (GNPs) in the epoxy polymer were likely to be considered.

#### 3.2.2. Tensile Properties

**Figure 8:**
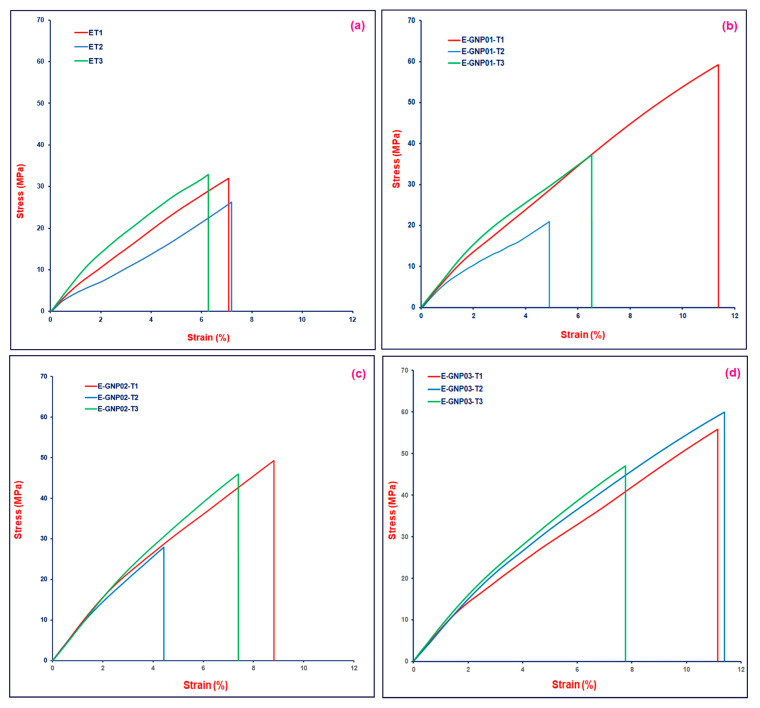
Tensile stress–strain curves of various polymer matrix composite samples. (**a**) Neat epoxy (ET), (**b**) E-GNP01-T composite samples, (**c**) E-GNP02-T composite samples, and (**d**) E-GNP03-T composite samples.

The tensile strength properties of epoxy/GNP nanocomposite structures were determined, and the results were averaged. Table 5 presents the tensile strength values of neat epoxy (ET) and the E-GNP01-T, E-GNP02-T, and E-GNP03-T composite structures. Figure 8 represents the stress–strain curves from the tensile test for pristine epoxy and the epoxy/GNP nanocomposites. The curves of test repetitions are presented in Figure 8 with assorted colors on each graph. The definition of the relevant curve is shown on the graph using sample codes.

##### Tensile Strength

**Figure 9:**
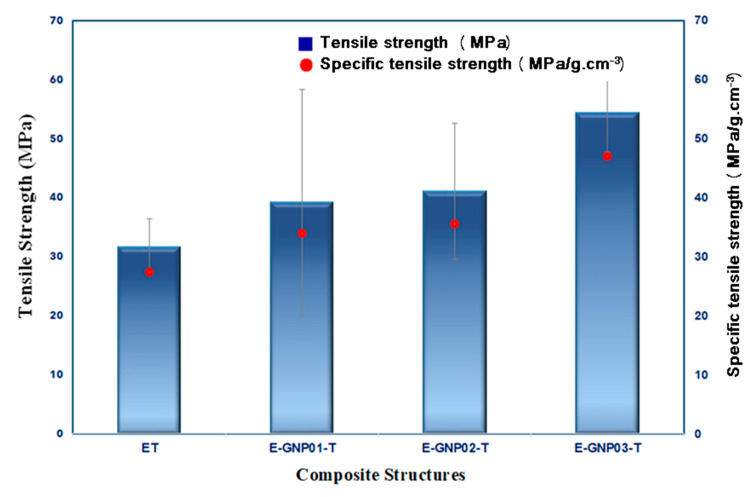
Tensile strength of control epoxy (ET) and epoxy/GNP nanocomposites with various GNP loading percentages (E-GNP01-T, E-GNP02-T, and E-GNP03-T).

Figure 9 exhibits the average tensile strength values of all the developed pristine epoxy and GNP-added epoxy nanocomposites with various filler loading percentages. The specific strength of a material is calculated by dividing its strength (force per unit area at failure) by its density, which is mostly used to compare materials. The specific tensile strengths of neat epoxy, E-GNP01-T, E-GNP02-T, and E-GNP03-T were 27.41 MPa/g-cm^−3^, 33.94 MPa/g-cm^−3^, 35.57 MPa/g-cm^−3^, and 47.01 MPa/g-cm^−3^, respectively. The tensile results revealed that the specific tensile strength was improved by adding a low content of GNPs compared to the neat epoxy. Incorporating GNPs into the epoxy matrix increased the tensile strength of E-GNP01-T, E-GNP02-T, and E-GNP03-T nanocomposites by 23.82%, 29.83%, and 71.62%, respectively, over neat epoxy. It was found that by incorporating only 0.3 wt.% GNPs into the neat epoxy system, the average tensile strength of the composites was significantly improved by 71.62% compared to virgin epoxy. The relatively small deviation in the mechanical properties of sample E-GNP02-T, in comparison to E-GNP01-T and E-GNP03-T, indicates a lower level of agglomeration and a more even dispersion of the nanofiller inside the matrix. The experimental data revealed that using GNPs as a nanofiller has a pronounced effect on the tensile properties of the epoxy composites. The interfacial adhesion between inclusions and the resin matrix significantly influences the tensile strength of polymer composites filled with inorganic particles. The load transfer properties are the most crucial factor in ensuring that the composite system works well. The interfacial strength between these components is essential, as the reinforcing function of GNPs relies on effective load transfer from the matrix to the GNPs. Enhanced mechanical properties arise from increased interaction between surface areas and the matrix, facilitating more substantial load transfer among distinct GNPs and the polymer matrix. Furthermore, adding GNPs improves tensile load-carrying performance by considering various fracture mechanisms. This probably included GNP layer shearing, layer separation in front of the crack tip, and crack deflection and bifurcation between the GNP and the matrix behind the crack tip. Additionally, plastic voids and shear hackles between GNPs and the epoxy can be critical during tensile load strength performance. Kilic et al. [72] examined the tensile properties of ductile and brittle epoxy polymers that have been strengthened with graphene nanoplatelets (GNPs) using different dispersion methods. However, the ductile epoxy with 1 wt.% GNPs exhibited a significant improvement in tensile strength, with an increase of up to 41% and a 19% rise in tensile modulus.

##### Tensile Modulus

**Figure 10:**
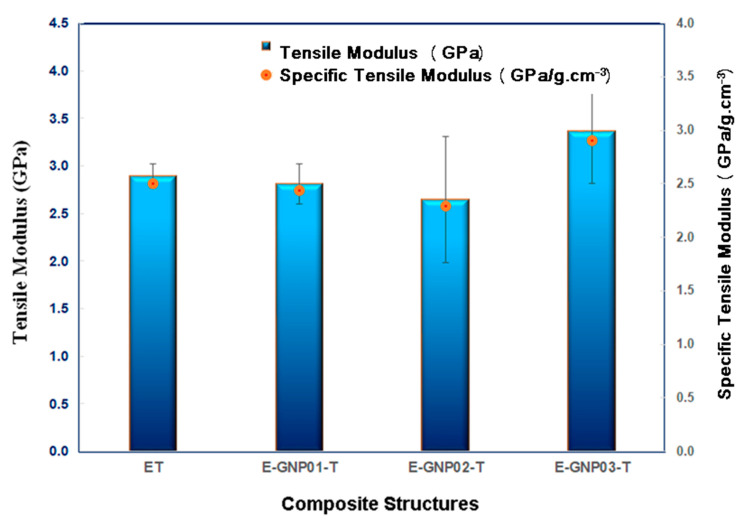
Tensile modulus of neat epoxy (ET) and epoxy/GNP nanocomposites with various filler loading percentages (E-GNP01-T, E-GNP02-T, and E-GNP03-T).

Figure 10 illustrates the average tensile modulus values for all the developed neat epoxy and epoxy/GNP nanocomposites with different loading percentages. Tensile test outcomes indicate an enhancement in the tensile modulus when GNPs were added compared to neat epoxy. A noticeable trend is observed, wherein the tensile modulus of the three composite systems increases as the weight fraction of GNPs rises. This implies that GNPs have a considerable stiffening impact on the epoxy matrix. As shown in Table 5, the tensile modulus of neat epoxy is 2.89 GPa, while for epoxy/GNP nanocomposites, it varies between 2.65 GPa and 3.36 GPa. Adding GNPs to the epoxy matrix slightly reduces the tensile modulus of E-GNP01-T and E-GNP02-T nanocomposites by 2.69% and 8.39%, respectively, compared to neat epoxy. This tensile modulus reduction may be due to minor GNP agglomerations and strain loosening at the boundary of the GNPs and epoxy. However, with 0.3 wt.% of GNPs in epoxy composites (E-GNP03-T), there is an enhancement of about 16.30% (from 2.89 GPa to 3.36 GPa) compared to neat epoxy. Incorporating higher GNP loading percentages increases the tensile modulus of the epoxy composites.

Introducing inorganic particles into polymer materials establishes a distinct skeleton within the matrix, potentially restricting molecular chain movement through bonding between GNPs and the matrix. This phenomenon enhances the stiffness of the composite system. Polymer composite stiffness rises with the increasing contribution of graphene nanoplatelets, particularly under conditions of homogeneous dispersion and optimal bonding within the matrix [73].

##### Tensile Strain

**Figure 11:**
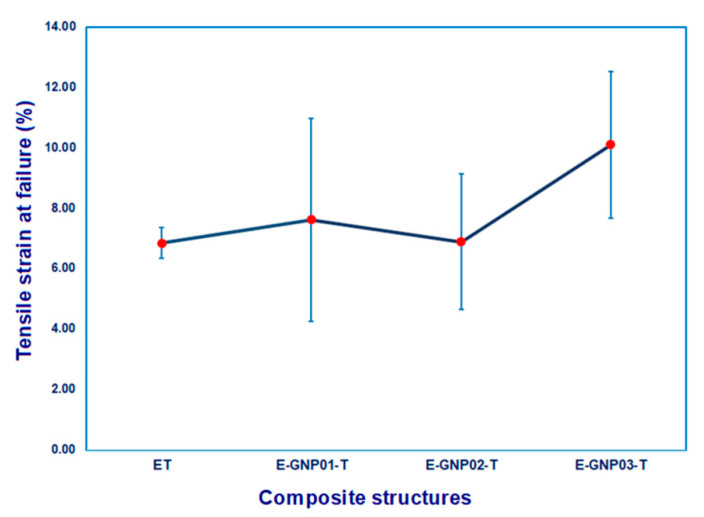
Tensile strain (%) values of pristine epoxy (ET) and GNP-added epoxy nanocomposites with different filler loading percentages (E-GNP01-T, E-GNP02-T, and E-GNP03-T).

Figure 11 depicts the correlation between tensile strain (%) and the weight fraction of GNPs in the epoxy/GNP composites. Tensile strain values, as presented in Table 5, for all developed neat epoxy and epoxy/GNP nanocomposites with different loading percentages, applied using an extensometer during tensile testing, ranged from 6.85% to 10.10%. A significant increment is found in tensile strain (%) after adding GNPs, in particular a 0.3% increase in loading rate for the epoxy matrix. The tensile strain of the E-GNP01-T, E-GNP02-T, and E-GNP03-T nanocomposites increased by 11.09%, 0.44% and 47.45%, respectively, compared to pure epoxy. As shown in Figure 11, the tensile strain values of epoxy/GNP composites were higher than those of pristine epoxy. This can be attributed to the stress concentration around graphene nanoplatelets, which may cause extra stretches (strain) as a form of plate-to-plate shearing, separation, deflection as a part of bifurcations that delayed catastrophic failure, and shear hackles were observed at the interface between epoxy and GNPs, indicating angular deformation due to localized delamination. This is likely caused by the sliding of graphene nanoplatelets within the polymer epoxy matrix during tensile load transfer. It can be concluded that adding GNPs as a nanofiller to epoxy composites improves their tensile strength, modulus, and tensile strain. Graphene nanoplatelets serve as effective reinforcing agents in nanocomposites, contributing to the augmentation of stiffness, strength, and elongation as the filler content rises.

#### 3.2.3. Compressive Properties

**Figure 12:**
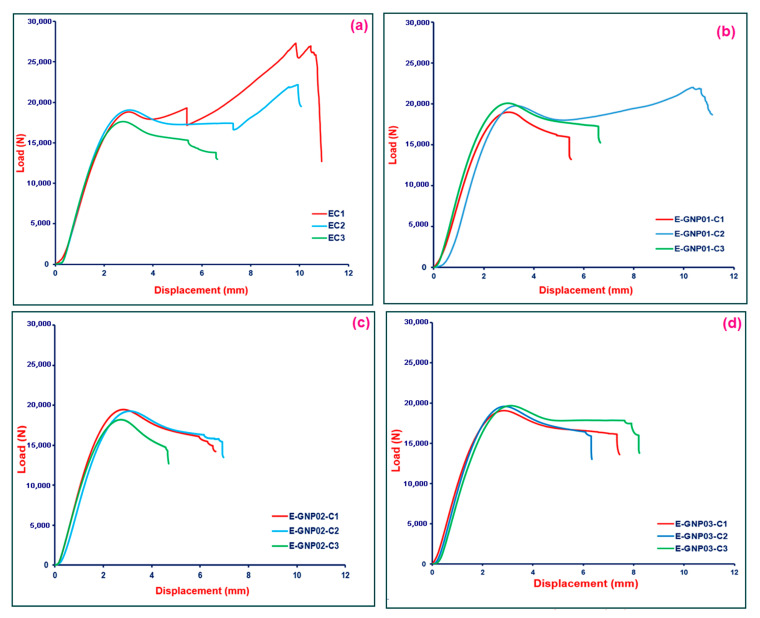
Compressive load–displacement graphs of the polymer matrix composite samples. (**a**) Pristine epoxy (EC), (**b**) E-GNP01-C composite samples, (**c**) E-GNP02-C composite samples, and (**d**) E-GNP03-C composite samples.

In this study, an effort was undertaken to explore the compressive behavior of polymer matrix nanocomposites. The compressive strength test results for neat epoxy (EC) and the E-GNP01-C, E-GNP02-C, and E-GNP03-C composite structures are presented in Table 6. Additionally, Figure 12 illustrates the compressive load–displacement curve for pristine epoxy and GNP-added epoxy nanocomposites. The curves of test repetitions are presented in the figure, with assorted colors on each graph. The definition of the relevant curve is shown on the graph using sample codes.

##### Compressive Strength

**Figure 13:**
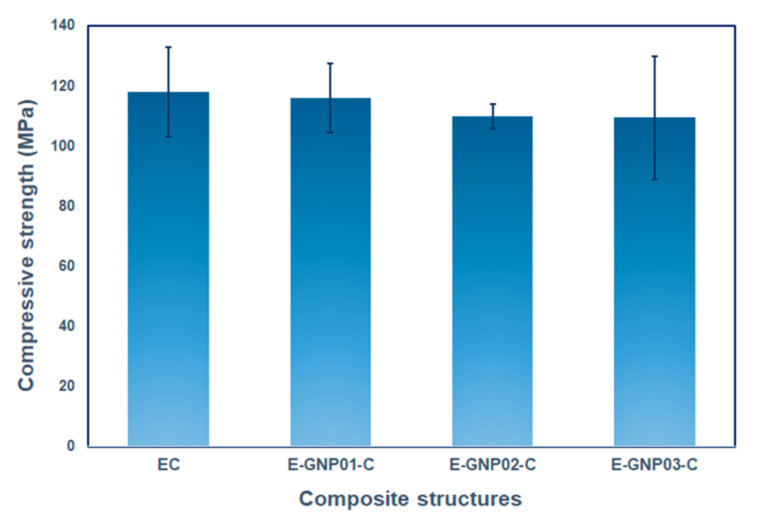
Compressive strength values of pristine epoxy (EC) and epoxy/GNP nanocomposites with different filler loading percentages (E-GNP01-C, E-GNP02-C, and E-GNP03-C).

Figure 13 depicts the compressive strengths of all the neat epoxy and epoxy/GNP nanocomposites with various filler loading percentages. The compressive results revealed that the compressive strength was slightly reduced by adding a low content of GNPs compared to the pristine epoxy. In Table 6, the average compressive strength of the neat epoxy composites is 117.97 MPa, whereas the average tensile strength values of the E-GNP01-C, E-GNP02-C, and E-GNP03-C nanocomposites are 116.10, 109.78, and 109.44 MPa, respectively. The compressive strength gradually reduced as GNPs loading percentages increased. Incorporating GNPs into the epoxy matrix reduced the compressive strength of the E-GNP01-C, E-GNP02-C, and E-GNP03-C nanocomposites by 1.59%, 6.94%, and 7.23%, respectively, compared to neat epoxy. One probable reason is that epoxy showed severe plastic yielding as a form of flexural buckling, which led to complete catastrophic axial splitting parallel to the compressive axis in the middle of the sample (Figure 16c,d). At the same time, this triggered an angular shear crack in the through-the-thickness that prevented smooth load distribution to the district graphene nanoplatelet and resulted in a premature fracture, which reduced the compressive strength. Additionally, local plastic voids at the GNP–epoxy boundary and multiple shear hackles can be considered critical during compressive load strength performance. To enhance the compressive strength of GNP nanocomposites, achieving the uniaxial distribution of axial splitting in both in-plane and out-of-plane directions within the structure is recommended, probably due to effective off-axis GNPs layer shearing and separations along with unit cell deformation causing a dampening effect. This can be achieved by incorporating large-sized graphene nanoplatelets that are homogeneously and multiaxially dispersed in the matrix. Kesavulu et al. [74] conducted a study where they dispersed varying weight percentages of alumina (Al_2_O_3_)-graphene nanoplatelets in an epoxy matrix. The objective was to enhance epoxy nanocomposites’ compressive performance and thermal stability. Adding 2 wt.% Al_2_O_3_ and 0.5 wt.% GNPs to the epoxy resulted in a considerable increase of 107.3% in compressive strength and 25.3% in compressive modulus compared to the pure epoxy samples.

##### Compressive Strain

**Figure 14:**
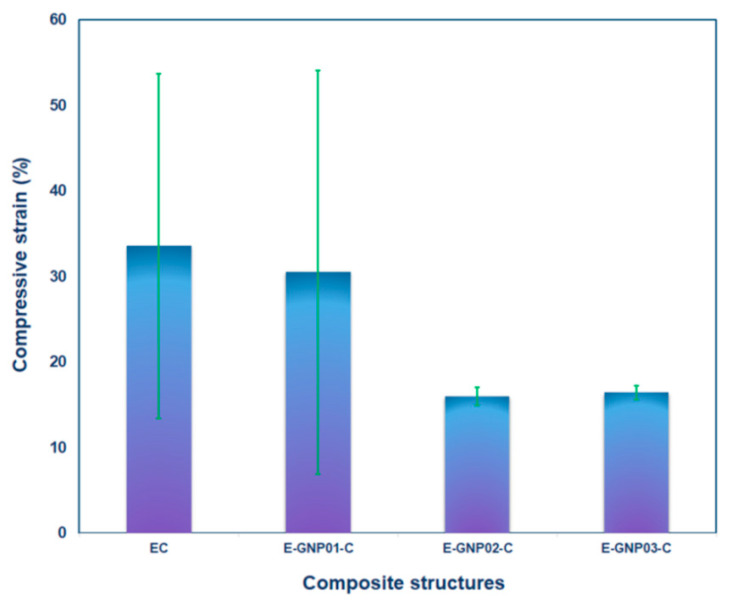
Compressive strain (%) of pristine epoxy (EC) and GNP-added epoxy nanocomposites with different filler loading percentages.

The compressive strain of all neat epoxy and epoxy/GNP nanocomposites with various loading percentages ranged from 33.52% to 15.97%, as presented in Table 6. There is a decrease in compressive strain (%) after adding GNPs to the epoxy matrix. The compressive strain of E-GNP01-C, E-GNP02-C, and E-GNP03-C nanocomposites was reduced by 9.75%, 52.35% and 51.04%, respectively, as shown in Figure 14. The mechanical reaction of epoxy changed from ductile to brittle with the addition of GNPs. Under compressive loading, this transition was shown to be more severe when considering complex toughening mechanisms including plastic yielding, brittle axial splitting, angular shear deformation, and plastic flow as a form of flexural buckling ((Figure 17b1,b2 and d2,d3), Figure 18c,d).

### 3.3. Fracture Morphology

#### 3.3.1. Tensile Fracture

**Figure 15:**
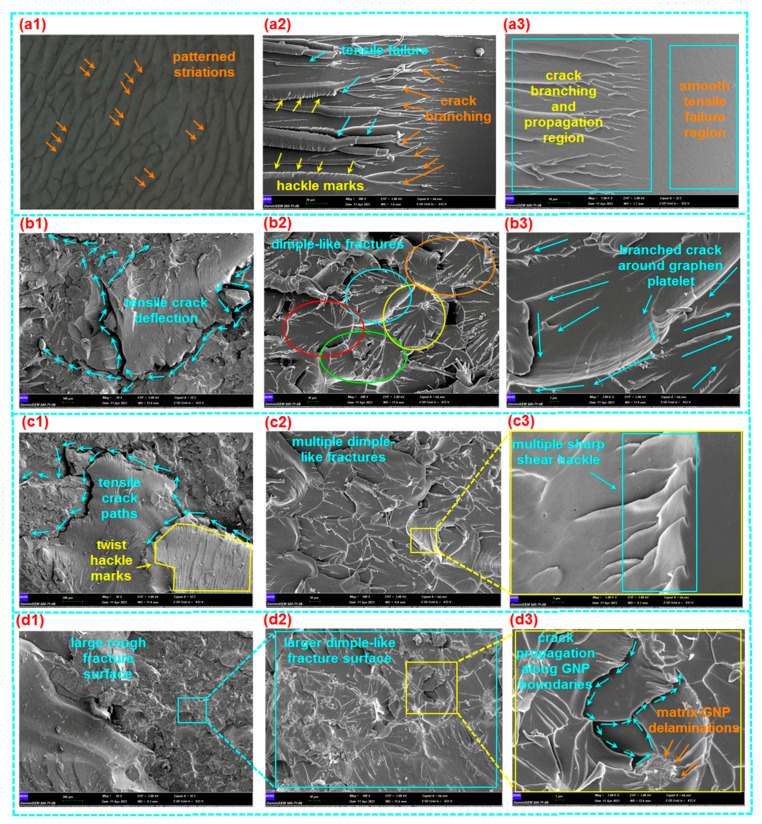
(**a1**) Top surface of neat epoxy (digital image), (**a2**,**a3**) fracture surface of neat epoxy in cross-section (SEM, scale: 20 µm, 10 µm; magnification: 500×, 1000×, respectively), (**b1**–**b3**) fracture surface of sample E-GNP01-T in cross-section (SEM, scale: 100 µm, 10 µm, 2 µm; magnification: 50×, 500×, 2000×, respectively), (**c1**–**c3**) fracture surface of sample E-GNP02-T in cross-section (SEM, scale: 200 µm, 10 µm, 1 µm; magnification: 50×, 500×, 5000×, respectively), and (**d1**–**d3**) fracture surface of sample E-GNP03-T in cross-section (SEM, scale: 200 µm, 10 µm, 2 µm; magnification 50×, 500×, 2000×, respectively).

**Figure 16:**
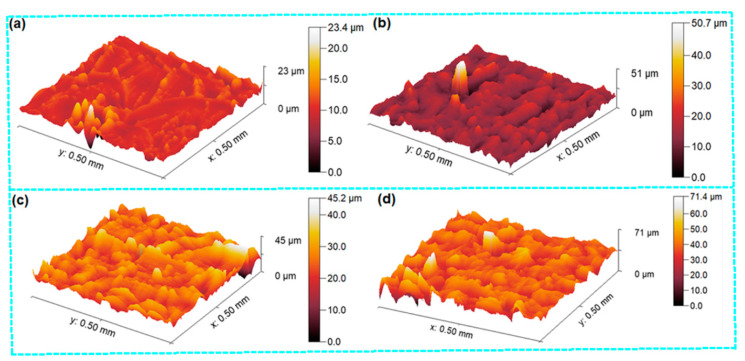
Failed surface roughness of (**a**) pristine epoxy (ET), (**b**) epoxy/0.1 wt.% GNP nanocomposite (E-GNP01-T), (**c**) epoxy/0.2 wt.% GNP nanocomposite (E-GNP02-T), and (**d**) epoxy/0.3 wt.% GNP nanocomposite (E-GNP03-T).

The tensile fracture morphology of various loaded GNPs (0.1 wt.%, 0.2 wt.%, and 0.3 wt.%) in polymer epoxy composites (E-GNP01-T, E-GNP02-T, and E-GNP03-T) is displayed in Figure 15a1–d3. In addition, Figure 16a–d exhibits the fracture surface roughness of epoxy/GNP composites. It was found that the surface of neat epoxy had patterned striations based on flow patterns before tensile loading was applied, as exhibited in Figure 15a1. The fracture surface of the virgin epoxy (ET) in the cross-section normal to the tensile loading axis (Figure 15a2) exhibited multiple collections of small crack propagations that converged as a kind of large, stretched, elliptical-shaped fracture. Moreover, the cross-section of the fractured epoxy was shown in two regions. The first region had a smooth, glassy surface on which catastrophic tensile failure was initiated and propagated perpendicular to the tensile axis (Figure 15a3). However, the second region had multiple collections of small crack propagations that converged to a large, stretched, elliptical-shaped fracture, probably due to the complex toughening mechanism of epoxy resin [32,75,76,77]. In addition, it was noted that the fracture boundaries had multiple shear hackle marks.

Multiple large tensile fractures with a deflected small crack during crack propagation in the FESEM image (magnification 50×) of sample E-GNP01-T were found, as exhibited in Figure 15b1. Further, multiple cracks propagated between the graphene nanoplatelets, producing dimple-like fractures (Figure 15b2). Here, the dimple fracture is recognized as a ductile fracture mechanism, progressing through three stages: void nucleation in the initial phase, void growth in the subsequent phase, and eventual coalescence leading to a catastrophic fracture [78]. The cracks that branched around the graphene nanoplatelets had multiple micro sharp shear hackle marks, as illustrated in the FESEM image (Figure 15b3).

It was identified that tensile crack propagation in sample E-GNP02-T was deflected and bifurcated around the graphene nanoplatelets (Figure 15c1). In addition, twist hackle marks [79] were found on epoxy/GNP nanocomposites. Multiple cracks on each graphene nanoplatelet boundary occurred as a form of dimple fracture with multiple randomly oriented hackle marks (Figure 15c2). Moreover, multiple sharp saw-shaped shear hackle marks with microvoids on the were identified epoxy–graphene nanoplatelets (Figure 15c3). Various local plastic voids [32,80] were also identified. They could occur in GNPs and matrix boundaries (Figure 15c3,d3).

In the FESEM micrograph, a large rough fracture surface was found on sample E-GNP03-T, probably because of the increasing GNP uploading rate in the epoxy resin (Figure 15d1). Numerous dimple-like fracture surfaces were also observed (magnification 500×, Figure 15d2). Local matrix breakages and delamination between epoxy and graphene nanoplatelets were observed (Figure 15d3). Moreover, crack propagations along GNP boundaries were found, probably leading to frictional-based in-plane pullout. This may cause layer-to-layer graphene nanoplatelet shear deformation and break the weak Van der Walls bonding force. Therefore, new crack fronts were created.

Surface roughness measurements on tensile fractured samples were performed on the best-represented failed area of their cross-sections by 500 µm × 500 µm. It was found that the fractured surface roughness (root mean square, RMS, roughness), especially for branching cracks, and propagation region of neat epoxy (ET) was 1.394 micron and the maximum roughness was about 23 micron (Figure 16a). On the other hand, the surface roughness values of E-GNP01-T, E-GNP02-T, and E-GNP03-T were 3.766 micron, 4.070 micron, and 5.637 microns, respectively. Their maximum roughness values were 51 micron, 45 micron, and 71 micron, respectively (Figure 16b–d). These findings demonstrate that as the loading rates of GNPs increased from 0.1 wt.% to 0.3 wt.%, there was a corresponding rise in surface roughness of approximately 49.68%.

The main primary fracture mechanism of epoxy/GNP nanocomposites is that delamination occurs between the interface regions of the epoxy and GNPs before or after brittle matrix breakages due to increased tensile stress levels. After that, cracks propagate around graphene nanoplatelets, causing crack bridging. Then, graphene nanoplatelets engage in pullout, which likely leads to in-plane bending deformation on platelets, indicating layer-to-layer deformed intertwined concave-shaped surfaces, as shown in Figure 15d3. Therefore, the fracture surface and surface roughness increased due to the graphene nanoplatelets in the polymer epoxy resin. Consequently, the tensile strength of GNP-added epoxy nanocomposite increased depending on the GNP loading rate. On the other hand, failure mode characteristics were changed from glassy sharp breakages in neat epoxy to rough brittle breakages in epoxy/GNP nanocomposites due to several mechanisms involved, including large fracture surfaces, large surface roughness [81], torturous crack deflection [55], bifurcate paths, graphene nanoplatelet bending [82], and probably multilayer plate separations [83].

#### 3.3.2. Compressive Fracture

**Figure 17:**
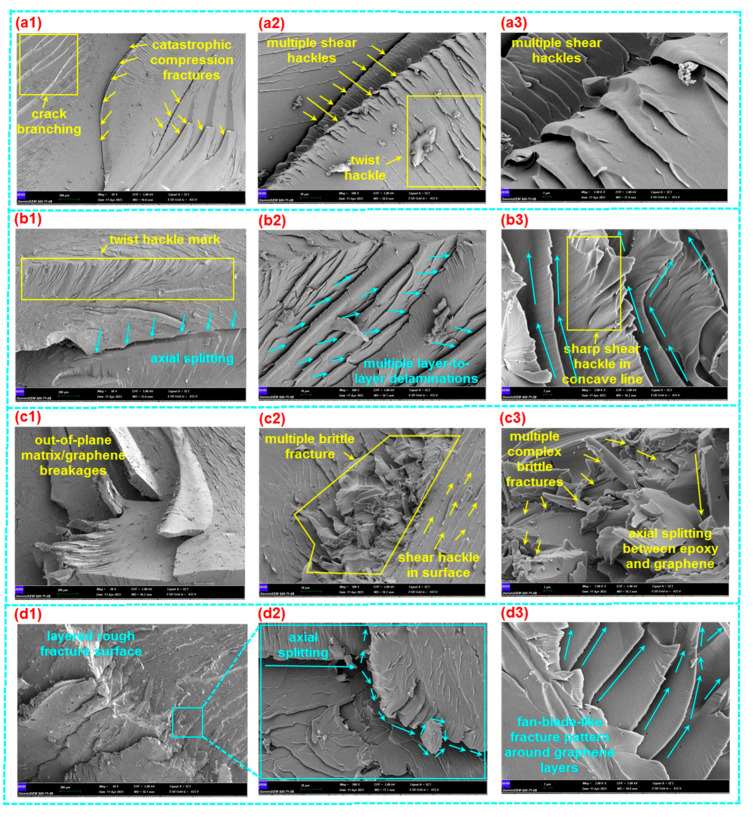
(**a1**–**a3**) Fracture surface of neat epoxy (EC) in parallel to compression axis (SEM, scale: 200 µm, 20 µm, 2 µm; magnification 50×, 500×, 2000×, respectively), (**b1**–**b3**) fracture surface of sample E-GNP01-C in parallel to compression axis (SEM, scale: 200 µm, 10 µm, 2 µm; magnification 50×, 500×, 2000×, respectively), (**c1**–**c3**) fracture surface of sample E-GNP02-C in parallel to compression axis (SEM, scale: 200 µm, 20 µm, 2 µm; magnification 50×, 500×, 2000×, respectively) and (**d1**–**d3**) fracture surface of sample E-GNP03-C in parallel to compression axis (SEM, scale: 200 µm, 20 µm, 2 µm; magnification 50×, 500×, 2000×, respectively).

The compressive fracture morphologies of various loaded GNPs (0.1 wt.%, 0.2 wt.%, and 0.3 wt.%) in polymer epoxy nanocomposites (E-GNP01-C, E-GNP02-C, and E-GNP03-C) are shown in Figure 17a1–d3. The failed surface of the neat epoxy (EC) parallel to the compressive loading axis displayed large axial splitting with multiple collections of small crack propagations (Figure 17a1). Further, fracture surfaces had multiple shear and twisted hackle marks (Figure 17a2,a3).

A single large-size axial splitting fracture [84] with various deflected small-size twist hackles around the splitting line in the FESEM image (magnification 50×) of sample E-GNP01-C was observed, as exhibited in Figure 17b1. Moreover, multiple cracks propagated between the graphene nanoplatelets producing adjacent layer-to-layer delamination (Figure 17b2), in which sharp shear hackles in the fracture surfaces were observed. Delamination exhibited a concave crack propagation line, probably due to the plastic yielding and generating flexural-torsional buckling under compressive loading (Figure 17b3).

It was observed in the FESEM micrograph that several out-of-plane matrix/graphene breakages and multiple adjacent in-plane cracks in sample E-GNP02-C were observed (Figure 17c1). In addition, multiple complex brittle fractures, shear hackles in the fractured surface, and axial splitting between epoxy and graphene nanoplatelets were identified (Figure 17c2,c3). Additionally, several local plastic voids [32,80] were observed. They could occur in GNPs and matrix boundaries (Figure 17c3,d3). A sizeable rough fracture surface in sample E-GNP03-C was obtained, probably because of the increasing graphene uploading rate in the epoxy resin (Figure 17d1). Further, axial splitting and diverted cracks around GNPs in the epoxy were observed (magnification 500×, Figure 17d2). Fan-blade-like local matrix breakages and delamination between the epoxy and graphene nanoplatelets were found (Figure 17d3). Moreover, multiple crack propagations along GNP boundaries were found, probably leading to frictional-based in-plane pullout. This may cause layer-to-layer graphene nanoplatelets to undergo angular shear deformation, and new crack fronts will probably be created.

In general, the primary fracture mechanism of the epoxy/GNP nanocomposites is that delamination occurs between the interface regions of the epoxy and GNPs before or after brittle matrix breakages due to increased compressive stress level. Subsequently, the crack propagates around the graphene nanoplatelets, deflecting the in-plane and out-of-plane areas within the structure. At the same time, this leads to out-of-plane flexural-torsional buckling deformation. On the other hand, the failure modes of the GNP-added structures were mainly plastic yielding/plastic flexural buckling, and axial splitting where crack propagation was deflected and appeared as a brittle angular shear crack in the through-the-thickness direction. Further study is required on the effect of off-axis graphene nanoplatelet position in epoxy/GNP nanocomposites.

**Figure 18:**
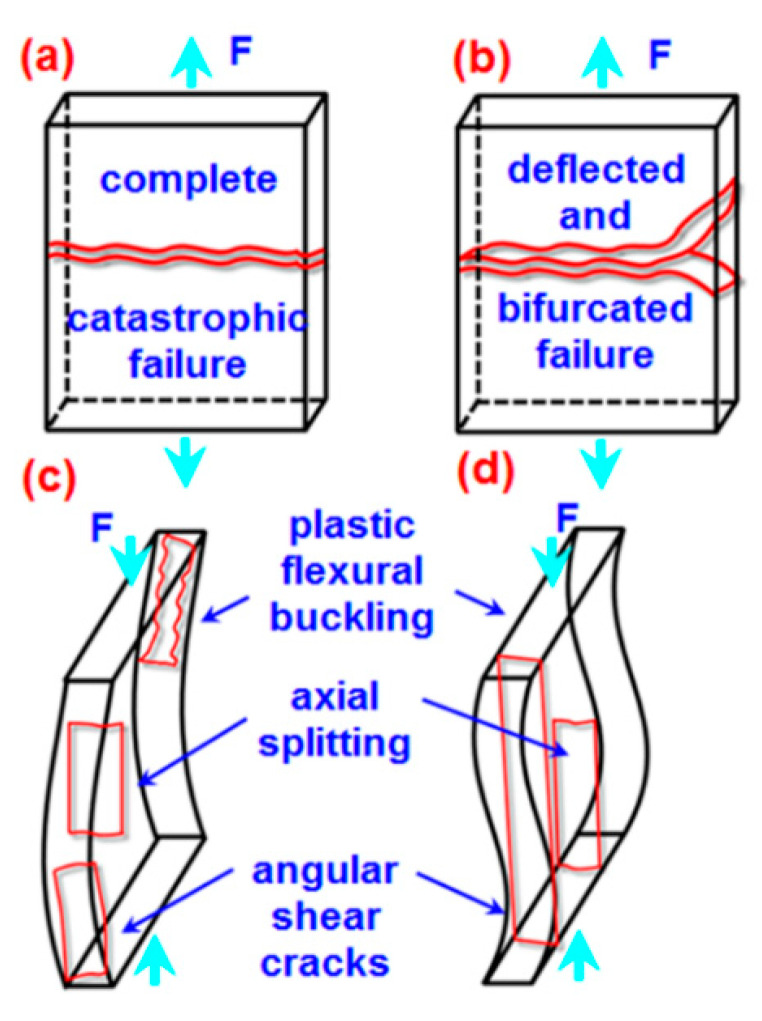
The failure mode of epoxy/graphene nanoplatelet nanocomposite structures in macroscale. (**a**) Tensile failure in a neat epoxy structure, (**b**) tensile failure in an epoxy/GNP structure, (**c**) compressive failure in a neat epoxy structure, (**d**) compressive failure in an epoxy/GNP structure.

The tensile and compressive macro-scale failure modes of epoxy/GNP nanocomposites are schematically exhibited in Figure 18a–d. In tensile failure, sharp and complete glassy fractures perpendicular to the tensile axis of pure epoxy structure occurred (Figure 18a). In contrast, a few deflected and bifurcated rough and brittle fractures in the epoxy/GNP nanocomposite were observed, as shown schematically in Figure 18b, respectively. Likely, the polymer epoxy distributed complex stress to numerous graphene nanoplatelets with large surface areas, enhancing the tensile load-carrying performance of the nanocomposite through various fracture toughness mechanisms. These mechanisms include crack deflection, bifurcation, multiple plate separations, biaxial plate pullout [85], and friction from rough fracture surface areas.

In compressive failure, plastic flexural buckling (inelastic deformation), an angular shear crack in the through-the-thickness at the boundaries, and the almost complete catastrophic axial splitting parallel to the compressive axis of the middle of the pure epoxy structure occurred (Figure 18c). Similarly, plastic flexural buckling (inelastic deformation), an angular shear crack in the through thickness near boundaries, and brittle axial splitting parallel to the compressive axis of the epoxy/GNP structure were observed, as exhibited schematically in Figure 18d. Results from tensile and compressive macro-scale failure modes indicated that epoxy/GNP nanocomposites behaved as damage-tolerant materials. On the other hand, when the loading direction changed from tensile to compressive, this created almost entirely different failure modes, from dimple-like to uniaxial splitting fractures. In this respect, future studies on nanoparticle-added materials are required, especially for their compressive strength, stiffness, and failure properties.

### 3.4. Thermal Conductivity

Enhancing thermal conductivity achieved by incorporating nanofillers into a thermally inefficient matrix is crucial. Dispersion, loading ratios, and, most critically, the temperature resistance of the polymer–nanofiller interface all influence conductivity. GNPs have been promising nanoscale conductive fillers since they were first combined the 2D effective layered structure a few years ago. The thermal conductivity of epoxy composites was assessed by incorporating varying loading percentages of GNPs (0.1 wt.%, 0.2 wt.%, and 0.3 wt.%). Factors influencing composite thermal conductivity encompass filler type, loading level, dispersion, particle characteristics (shape, alignment, waviness), and thermal contact resistance at the matrix–nanofiller interface [86].

**Figure 19:**
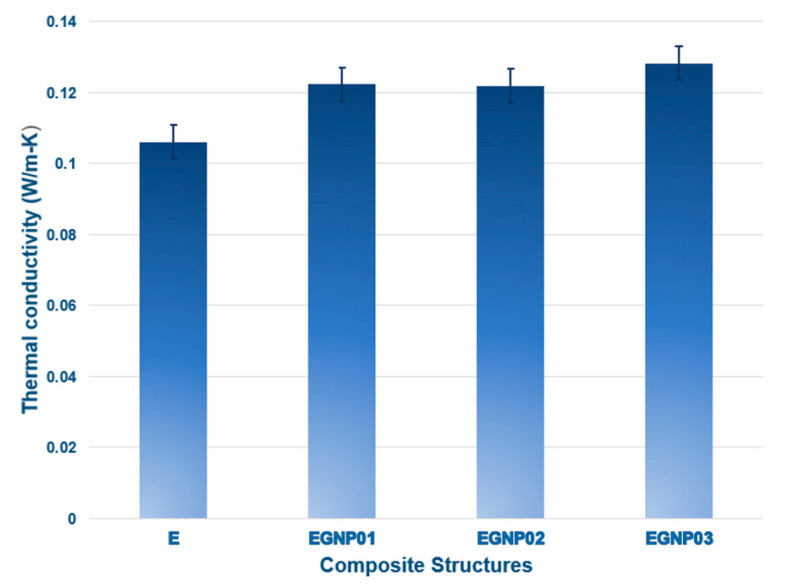
Thermal conductivity values of neat epoxy and GNP-added epoxy nanocomposites with different filler loading percentages (E-GNP01, E-GNP02, and E-GNP03).

Understanding the thermal conductivity of graphene in polymers is a multifaceted phenomenon, given the substantial surface area per unit volume possessed by graphene. When a polymer is added to graphene, many interfaces are created. The interface will result from the scattering of phonons and the introduction of ultrahigh interfacial thermal resistance. The graphene–polymer interface makes it difficult for heat to pass through. In many studies, the molecular chains of polymers and graphene’s surface molecular chains have been considered to form an interlayer. This interlayer’s intertwined molecular chains effectively minimize interfacial phonon scattering and thermal resistance at the interface. Thermal conductivity increases when graphene in composites directly interacts with a heat source. Creating a composite with enhanced thermal conductivity involves augmenting thermal pathways and minimizing resistance between graphene and the graphene–polymer interface. GNPs, when incorporated into polymer matrices, promote closer platelet interaction, diminishing the thin epoxy layer between nanofillers. This reduction mitigates phonon dispersion arising from the contrast in thermal conductivity between the nanofillers and the insulating epoxy matrix. Additionally, GNPs offer 2D channels for efficient phonon transport. This is because adding flat-shaped GNPs to epoxy resins reduces the viscosity of the resin [77]. GNPs exhibit a two-dimensional (2D) planar structure conducive to efficient phonon transport. Their expansive specific surface area facilitates substantial surface contact with the polymer, enhancing the composites’ thermal conductivity [87]. Figure 19 illustrates the thermal conductivity test results for neat epoxy and epoxy/GNP nanocomposites at varying ratios. The findings indicate improved thermal conductivity when GNPs are added to epoxy composites. Adding GNPs to epoxy nanocomposites improved them by 15.27%, 14.89%, and 20.92% for the E-GNP01, E-GNP02, and E-GNP03 nanocomposites. With the incorporation of 0.3 wt.% GNPs into epoxy, the maximum thermal conductivity reached 0.1283 W/m-K, marking a 20.92% advancement compared to neat epoxy (0.1061 W/m-K). GNPs are electrically and thermally conductive principally because of the electron’s presence. The high aspect ratio of the GNP filler could also play a role in the thermal transport mechanism The consistent enhancement in thermal conductivity within the composites is attributed to the even dispersion of nanofillers in the polymer matrix, facilitating efficient heat propagation primarily through electron movement through percolative paths. This steady increase in thermal conductivity is sustained by the uniform dispersion and network formation of nanofillers in the polymer matrix. Wang et al. [28] observed a significant enhancement in thermal conductivity when bigger GNPs were added, reaching a 115% increase at a loading of 5 wt.%.

## 4. Conclusions

By incorporating only 0.3 wt.% GNPs into the neat epoxy system, the average tensile strength, tensile modulus, and tensile strain of the composites were significantly enhanced by 71.62%, 16.30%, and 47.45% compared to neat epoxy. The substantial surface area of graphene nanoplatelets may be responsible for enhancing the tensile load-carrying performance of the nanocomposite. This enhancement is achieved through crack deflection, a new fracture front, and increased friction from the rough fracture surface. Conversely, as GNP loading percentages increased, the compressive strength gradually diminished. This decline is likely attributed to severe plastic yielding in the epoxy, leading to axial splitting and angular shear cracks, causing premature fractures, and adversely affecting the compressive strength of the epoxy nanocomposite. Simultaneously, with the addition of 0.3 wt.% GNPs, the thermal conductivity peaked at 0.1283 W/mK, marking a 20.92% improvement over neat epoxy (0.1061 W/mK). The consistent enhancement in thermal conductivity is credited to the uniform dispersion of nanofillers in the polymer matrix, promoting efficient heat propagation, primarily through phonon diffusion in the GNPs.

The tensile failure mode in neat epoxy exhibited sharp and catastrophic glassy fractures, whereas deflected and bifurcated rough and brittle dimple-like fractures in epoxy/GNP nanocomposite were found. Several fracture toughness mechanisms were identified, including crack deflection and bifurcation, multiple plate separations, probably biaxial plate pullout, and friction due to rough fracture surface areas. On the contrary, the compressive failure mode in pure epoxy showed plastic flexural buckling, angular shear cracks, and almost catastrophic axial splitting. Likewise, plastic yielding flexural buckling and large brittle axial splitting, including angular shear cracks in the through thickness near the boundaries of the epoxy/GNP structure, were observed.

Results from tensile and compressive macroscale failure modes indicated that epoxy/GNP nanocomposites behaved as damage-tolerant materials. When the loading direction changed from tensile to compressive, this created almost entirely different failure modes, from dimple-like to uniaxial splitting fracture. Additional investigations are needed to delve into the compressive strength, stiffness, and failure properties of epoxy/GNP nanocomposites, with a specific emphasis on understanding the off-axis contribution of nanoparticles.

## Figures and Tables

**Table 1:** Specification of epoxy and hardener.

Supplier	Type	Density (g/cm^3^)	Viscosity (m Pas)	Operating Temperature (°C)
Epoxy Resin Dost Kimya Co, TR Hardener Dost Kimya Co, TR	A modified version of bisphenol A/F (MGS L326)	1.14–1.19	5000–7000	−60/+150
	A modified version of cycloaliphatic amines (MGS H265)	0.91–0.93	10–20	−60/+150

**Table 2:** Experimental design of epoxy/graphene nanoplatelet nanocomposites.

Sample Number	Composite Code	Sampling Type
01	E	Neat epoxy
02	E-GNP01	Epoxy/GNPs (0.1 wt.%)
03	E-GNP02	Epoxy/GNPs (0.2 wt.%)
04	E-GNP03	Epoxy/GNPs (0.3 wt.%)

**Table 3:** FTIR spectra identified for epoxy and GNPs added to epoxy composites.

SI No.	Vibrations	Wavenumber (cm^−1^)
01	–OH stretching	3381
02	C–H stretching	2800–3000
03	Asymmetrical stretching of –CH of –CH_2_ group	2928
04	Symmetrical stretching of –CH of –CH_3_ group	2868
05	C––C stretching bands of aromatic rings	1608
06	N–O stretching	1508
07	Epoxy ring mode	1294, 955, 875
08	C–O stretching of the aromatic ring	1180
09	Symmetrical aromatic C–O stretching	1035
10	Aromatic ring bent out of plane	826
11	C–OH, C–H, N–H bend	555

**Table 4:** The measured density of epoxy composite plates.

Sample Number	Sample Description	Composite Panel Code	Average Density (g/cm^3^)	Standard Deviation	Coefficient of Variation
01	Neat Epoxy	E	1.153 ± 0.002	0.0023	0.0020
02	Epoxy/GNPs (0.1 wt.%)	E-GNP01	1.153 ± 0.000	0	0
03	Epoxy/GNPs (0.2 wt.%)	E-GNP02	1.154 ± 0.001	0.0006	0.005
04	Epoxy/GNPs (0.3 wt.%)	E-GNP03	1.154 ± 0.000	0	0

**Table 5:** Tensile strength test results of all epoxy/GNP composites.

Sample Code	Tensile Load (Max.) (N)	Displacement (mm)	Tensile Strength (MPa)	Tensile Modulus (GPa)	Tensile Strain Extensometer (%)
ET	4980.50	4.11	31.61 ± 4.84	2.89 ± 0.13	6.85 ± 0.50
E-GNP01-T	7117.58	4.56	39.14 ± 19.21	2.81 ± 0.21	7.61 ± 3.36
E-GNP02-T	7626.10	4.13	41.04 ± 11.18	2.65 ± 0.66	6.88 ± 2.24
E-GNP03-T	10,247.78	6.06	54.25 ± 6.61	3.36 ± 0.55	10.10 ± 2.43

**Table 6:** Compressive strengths of different GNP-added epoxy nanocomposite plates.

Sample Code	Compressive Load (Max.) (N)	Compressive Displacement (mm)	Compressive Strength (MPa)	Compressive Strain (%)
EC	19,702.30	0.787	117.97 ± 15.00	33.52 ± 20.12
E-GNP01-C	20,348.20	0.787	116.10 ± 11.49	30.25 ± 23.59
E-GNP02-C	18,986.27	0.818	109.78 ± 4.07	15.97 ± 1.03
E-GNP03-C	19,438.30	0.899	109.44 ± 2.06	16.40 ± 0.8

## Data Availability

All data generated or analyzed during this study are included in this published article.
